# The Binding Ability of Mercury (Hg) to Photosystem I and II Explained the Difference in Its Toxicity on the Two Photosystems of *Chlorella pyrenoidosa*

**DOI:** 10.3390/toxics10080455

**Published:** 2022-08-06

**Authors:** Shuzhi Wang, Jia Duo, Rehemanjiang Wufuer, Wenfeng Li, Xiangliang Pan

**Affiliations:** 1 National Engineering Technology Research Center for Desert-Oasis Ecological Construction, Xinjiang Institute of Ecology and Geography, Chinese Academy of Sciences, 818 South Beijing Road, Urumqi 830011, China; 2 Xinjiang Key Laboratory of Environmental Pollution and Bioremediation, Xinjiang Institute of Ecology and Geography, Chinese Academy of Sciences, Urumqi 830011, China; 3 Key Laboratory of Microbial Technology for Industrial Pollution Control of Zhejiang Province, College of Environment, Zhejiang University of Technology, Hangzhou 310014, China

**Keywords:** mercury, binding ability, chlorophyll *a* fluorescence, P700^+^ absorbance change, quantum yield, electron transport rate, cyclic electron flow

## Abstract

Mercury (Hg) poses high toxicity to organisms including algae. Studies showed that the growth and photosynthesis of green algae such as *Chlorella* are vulnerable to Hg stress. However, the differences between the activities and tolerance of photosystem I and II (PSI and PSII) of green microalgae under Hg exposure are still little known. Responses of quantum yields and electron transport rates (ETRs) of PSI and PSII of *Chlorella* *pyrenoidosa* to 0.05–1 mg/L Hg^2+^ were simultaneously measured for the first time by using the Dual-PAM-100 system. The photosystems were isolated to analyze the characteristics of toxicity of Hg during the binding process. The inhibition of Hg^2+^ on growth and photosystems was found. PSII was more seriously affected by Hg^2+^ than PSI. After Hg^2+^ exposure, the photochemical quantum yield of PSII [Y(II)] decreased with the increase in non-photochemical fluorescence quenching [Y(NO) and Y(NPQ)]. The toxic effects of Hg on the photochemical quantum yield and ETR in PSI were lower than those of PSII. The stimulation of cyclic electron yield (CEF) was essential for the stability and protection of PSI under Hg stress and played an important role in the induction of non-photochemical quenching (NPQ). The results showed a strong combination ability of Hg ions and photosystem particles. The number of the binding sites (n) of Hg on PSII was more than that of PSI, which may explain the different toxicity of Hg on PSII and PSI.

## 1. Introduction

Heavy metal pollution is one of the most critical environmental problems, posing a serious threat to the ecosystem and human health [1,2]. Among the heavy metals, mercury (Hg) is one of the most toxic metals and is receiving increasing concern from the whole world [3,4,5]. China is one of the countries with the largest mercury production, consumption and anthropogenic emissions. However, the status of mercury pollution and its associated health risks in China are still relatively unknown [6]. Serious Hg^2+^ pollution was found in some lakes, rivers, and soil [7]. Although the Hg concentration in natural water is low, it may reach 0.69 μg/L in Chinese freshwater systems [8]. In heavily contaminated sites such as Wanshan, which was the largest Hg production area in China, Hg in water could be as high as 10,000 ng/L [8]. Hg in the environment poses a great risk to organisms including humans due to its non-biodegradability and biomagnification along the food web [9,10,11]. Extensive studies show the toxicity of Hg to photosynthetic apparatus [12,13,14]. Hg could lead to some serious biochemical and physiological disorders in organisms [15,16].

As human activities have increased the inputs of mercury into aquatic ecosystems over the past decades, it is necessary to investigate the toxicity of Hg to aquatic organisms [9]. Algae are the main entry point of Hg into the aquatic food web and play an important role in absorbing and transforming Hg species in the aquatic ecosystem [17]. Research on the physiological response of phytoplankton to Hg is of great significance for understanding the toxicity and risk of Hg to the aquatic ecosystem [9].

Many studies showed that Hg showed inhibition effects on growth [16], chlorophyll biosynthesis [18,19], and activity of photosynthesis of plants and phytoplankton [12,14,20]. Most studies investigated the adverse effects of Hg on photosystem II (PSII) activities, located in the donor and/or the acceptor sides and the reaction center of PSII of plants [21,22]. Some research found that photosystem I (PSI) activity could be reduced under the stress of Hg [12]. However, the effects of heavy metals on PSII and PSI are separately studied in most studies. Moreover, the differences between the activities and tolerance of PSII and PSI in intact algal cells under Hg exposure are also little known. Therefore, a Dual-PAM-100 system was used in the present study to reflect the physiological status of PSII and PSI under Hg toxicity, which showed its advantage in simultaneous measurements of chlorophyll *a* fluorescence and P700^+^ absorbance changes of intact cells [23,24]. In addition, some heavy metals were reported to stimulate the cyclic electron flow (CEF) around PSI [25,26]. These studies showed that CEF played an important role in coping with abiotic stress and protecting PSI. However, whether CEF was stimulated under Hg stress, and the response and physiological function of CEF around PSI under Hg stress still need further study.

In the present study, the toxic effects of inorganic mercury (Hg^2+^) on the activities of PSII and PSI of green microalga *Chlorella pyrenoidosa* were studied. The green microalgae, such as *C. Ppyrenoidosa* used in the study, are the main primary producers and essential in food chains in aquatic ecosystems, and *C. pyrenoidosa* has often been used as a model microbial species for examining the effects of contaminants on photosynthesis [25,27]. CEF around PSI and non-photochemical fluorescence quenching, which provided protection to photosynthetic apparatus under stress, were also tested to show the effects of Hg^2+^ on the regulation of electron transport and energy usage or dissipation. We assumed that the toxicity of heavy metals in photosynthesis was related to their binding abilities to photosynthetic apparatus, so the binding features of Hg ions and photosystem particles were detected to explain the different toxicity of Hg on PSII and PSI. The results will enhance the understanding of mercury toxicity and provide a basis for applications in bioremediation.

## 2. Materials and Methods

### 2.1. Culture of Chlorella pyrenoidosa

*Chlorella pyrenoidosa* (FACHB-9) was purchased from Freshwater Algae Culture Collection of Institute of Hydrobiology, Chinese Academy of Sciences (Wuhan, China). The cells were cultured in BG-11 medium [28]. All the cultures were carried out at 25 °C under 30 μmol photons m^−^^2^ s^−^^1^ with a 12:12 h light: dark cycle. The growth of cultures was monitored daily by testing optical density at 680 nm (OD_680_), which was positively correlated with algal cell density. OD_680_ was measured with a UV2800 spectrophotometer (Unico, Shanghai, China) to detect the growth phase of the cells.

### 2.2. Hg^2+^ Treatments

*C. pyrenoidosa* cells in exponential growth phase, with OD_680_ around 0.3 to 0.8, were harvested by centrifugation at 8000 r min^−1^ for 5 min, then the cells were resuspended and cultured in 50 mL flasks with 30 mL of BG-11 medium containing various concentrations (0, 0.05, 0.5 and 1 mg/L) of Hg^2+^. Hg^2+^ was applied in the form of analytical-grade HgCl_2_ and dissolved in BG-11 medium. Each treatment had three replicated samples. The samples with 0 mg/L Hg^2+^ were used as control. The OD_680_ of all the cultures was close to 0.3 just after onset of Hg^2+^ treatments. All treatments were performed under the same culture condition as described above. 

### 2.3. Measurement of Growth

Measurements were carried out after the exposure of the cells to various concentrations of Hg for 24 h. OD_680_ of all the cultures was measured. The specific growth rate (*μ*, d^−1^) of algae for the exposure duration (1 day) was calculated from the logistic equation according to Nam et al. (2009) [29].

### 2.4. Measurement of Pigments Content

After the cells were exposed to various concentrations of Hg^2+^ for 24 h, 3 mL of cells solution of each culture was harvested for extraction of pigments. The cells were collected by centrifugation at 8000 r min^−1^ for 5 min and then extracted in 80% acetone for 24 h in the dark. The contents of chlorophyll (Chl) *a*, *b*, and total carotenoids were determined by testing the absorbance of the supernatant (derived from centrifugation at 8000 r min^−1^ for another 5 min after extraction) with a UV2800 spectrophotometer (Unico, Shanghai, China) and then calculated in the formulae according to Lichtenthaler and Wellburn (1983) [30].

### 2.5. Measurement of Activities of PSII and PSI

Activities of PSII and PSI were measured using a dual-wavelength pulse-amplitude-modulated fluorescence monitoring system (Dual-PAM-100, Heinz Walz GmbH, Nürnberg, Germany). The Dual-PAM-100 system can detect P700^+^ absorbance changes and chlorophyll *a* fluorescence at the same time [31,32,33]. The sample collected for the measurement was adjusted to around 15 mg Chl *a*/L and then injected into the DUAL-K25 quartz glass cuvette supplied with the monitoring system. The cuvette was then sandwiched between the emitter head and detector head of the system. Each sample was dark-adapted for 5 min, and then saturation pulse and actinic light were applied according to the measurement routine [26]. Parameters were automatically calculated by the Dual-PAM-100 software during the measurement [34].

The minimal fluorescence after dark-adaptation (*F*_0_), the maximum fluorescence (*F*_m_) and the maximal change in P700^+^ signal (*P*_m_) were determined through application of saturation pulse. A typical experimental result obtained with Dual-PAM-100 is shown in Figure S2. The fluorescence intensity increased from *F*_0_ to *F*_m_. The variable fluorescence (*F*_v_) was calculated from *F*_0_ and *F*_m_ as: *F*_v_ = *F*_m_−*F*_0_. The maximal photochemical efficiency of photosystem II (PSII) was calculated as *F*_v_/*F*_m_ = (*F*_m_ − *F*_0_)/*F*_m_ [35]. The P700^+^ absorbance changes were detected by the application saturation pulse after far-red pre-illumination to determine *P*_m_. The saturation pulse transiently induces full P700 oxidation as shown in the increase in P700^+^ signal to its maximal value. P700 was reduced due to the electrons transported from PSII to PSI, and P700^+^ signal decreased. Briefly after the saturation pulse, the minimal P700^+^ signal (*P*_0_) was measured, indicating that P700 was fully reduced. The difference in P700^+^ signal between the fully reduced and oxidized states is denoted by *P*_m_ (Figure S2), which was the maximal signal of photo-oxidizable PSI reaction center (P700) and the indicator of the quantity of efficient PSI complex [31]. After the determination of *F*_0_, *F*_m_, and *P*_m_, rapid light response curves (RLCs) of quantum yields and electron transport rates (ETRs) was performed with the routine program of the Dual-PAM-100 software. During RLC mode, the actinic light was applied at each photosynthetic active radiation (PAR) for 30 s with increasing intensity (0, 13, 29, 60, 102, 173, 280, 437, 667, 1035 and 1601 μmol photons m^−^^2^ s^−^^1^) to conduct the rapid light response reaction. After each period of actinic light, the maximum fluorescence signal (*F*_m_′) and maximum P700^+^ signal (*P*_m_′) under actinic light were detected with the application of a saturation pulse. The P700^+^ signal (*P*) was recorded just before a saturation pulse. The minimum level of the P700^+^ signal (*P*_0_) was tested at a 1 s dark interval after each saturation pulse. The signals *P* and *P*_m_*’* were determined referring to *P*_0_.

The quantum yields of PSI and PSII were calculated automatically from the fluorescence and P700^+^ signals by the Dual-PAM software. The quantum yields of energy conversion in PSI were calculated according to Li et al. (2021) [32]:

Y(I) = (*P*_m_′ − *P*)/*P*_m_ (1)

Y(ND) = (*P* − *P*_0_)/*P*_m_ (2)

Y(NA) = (*P*_m_ − *P*_m_′)/*P*_m_ (3)

where Y(I) was effective photochemical quantum yield of PSI, Y(ND) was the quantum yield of non-photochemical energy dissipation in PSI reaction centers due to donor side limitation, and Y(NA) was the quantum yield of non-photochemical energy dissipation of PSI reaction centers due to acceptor side limitation. 

The quantum yields of energy conversion in PSII were calculated in the following equations [23,33]:

Y(II) = (*F*_m_′ − *F*)/*F*_m_′ (4)

Y(NPQ) = *F*/*F*_m_′ − *F*/*F*_m_ (5)

Y(NO) = *F*/*F*_m_ (6)

where Y(II) was the photochemical quantum yield of PSII, Y(NPQ) was the quantum yield of light-induced non-photochemical fluorescence quenching, and Y(NO) was the quantum yield of non-light-induced non-photochemical fluorescence quenching.

ETR in PSI [ETR(I)] and PSII [ETR(II)] were calculated and recorded by the Dual-PAM software [26,33]. Descriptive parameters of ETR(I) and ETR(II) during the light response reaction were derived by fitting RLCs to the exponential function [36,37]: *α*, the initial slope of RLCs of ETR(I) or ETR(II), which reflected the photon-capturing efficiency [38]; ETR_max_, the maximal electron transport rates in PSI or PSII; *I*_k_, the minimum saturating irradiance, which served as the index of light adaptation of PSI or PSII and calculated as ETR_max_/*α* [39].

### 2.6. Measurements of Quantum Yield of Cyclic Electron Flow

The quantum yield of CEF was calculated from the difference between Y(I) and Y(II) [40]:

Y(CEF) = Y(I) − Y(II)(7)

The ratios of Y(CEF)/Y(I), Y(II)/Y(I), and Y(CEF)/Y(II) were also calculated to show the change in the distribution of quantum yields between two photosystems. Y(CEF)/Y(II) indicated the ratio of quantum yield of CEF to that of linear electron flow (LEF) [40,41].

### 2.7. Isolation of Photosystems and the Binding with Hg Ions

Exponentially growing cells of *C. pyrenoidosa* were harvested by centrifugation for the isolation of PSI and PSII particles. Photosystems particles were isolated according to Cullen et al. (2007) [42] and Macro et al. (2019) [43]. PSI particles were resuspended in the PSI preservation solution (20 mM Tricine-NaOH, 10 mM NaCl, 10 mM KCl and 5 mM MgCl_2_; pH = 7.8). PSII particles were resuspended in the PSII preservation solution (0.4 M sucrose, 5 mM MgCl_2_, 10 mM NaCl, 40 mM MES-NaOH; pH = 6.5). The isolated PSI and PSII complexes were stored at −20 °C for next experiments in short term [42,43]. Samples were taken from the separated photosystem suspension for chlorophyll concentrations determination according to [44]. 

Three-dimensional (3D) fluorescence of two photosystems was determined using a fluorescence spectrophotometer (F-7000, Hitachi, Tokyo, Japan) at 298 K. The quenching effect of Hg on the fluorescence of two photosystems was detected to reflect the binding characteristic. Photosystems particles were resuspended in 0.05 M phosphate buffer (pH = 7.4) and adjusted chlorophyll concentration to 10 μg/mL. In total, 3 mL of suspension was added to the quartz cuvette for the measurement. Three-dimensional fluorescence spectroscopy showed the excitation peak was at 436 nm, which was consistent with the fluorescence of chlorophyll material at room temperature. PSI and PSII have emission peaks at the same position around 685 nm. So the fluorescence peak at EX436/EM685 was used to detect the quenching of the fluorescence of photosystems with Hg ions.

For the titration to test the quenching effects, 3 μL of 50 mM HgCl_2_ stock solution in distilled water was injected into the quartz glass cuvette and the concentration of Hg increased by 50 μM per titration. The solution was stirred for 15 min for equilibrium and the fluorescence at EX436/EM685 was recorded after each addition of Hg. The measurement was repeated 3 times. The equilibrium characteristics can be quantitatively described by the fitting parameters: association constant (Ka) and binding site (n), which were obtained from the Lineweaver–Burk equation as described by [45].

### 2.8. Statistics

Means and standard error (S.E.) were calculated for each treatment (n = 3). Analysis of Variance (one-way ANOVA) and Duncan’s test were performed to detect the significance of differences between different treatments (ANOVA, Duncan’s test, *p* < 0.05).

## 3. Results

### 3.1. Effects of Hg^2+^ on Growth Rates

**Figure 1:**
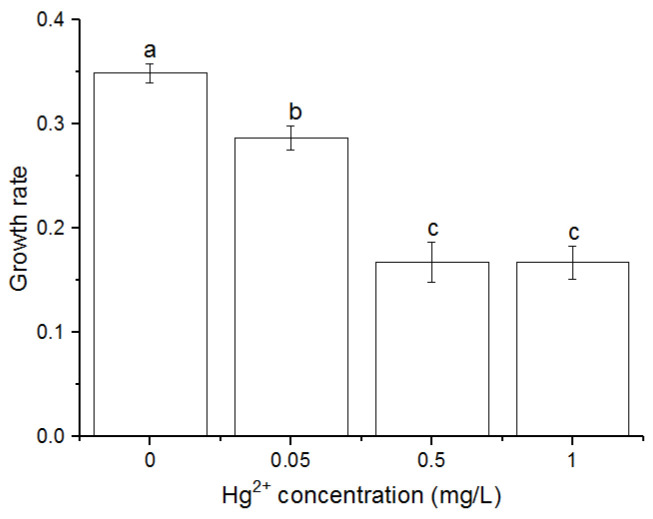
Specific growth rates (*μ*) of *Chlorella pyrenoidosa* cells after exposure to various concentrations of Hg^2+^ for 24 h. Data are means ± S.E. (n = 3). Significant differences between different treatments were shown as different letters (*p* < 0.05, ANOVA, Duncan’s test).

OD_680_ of the cells treated with Hg at various concentrations during the experiment were recorded (Figure S1). Exposure to Hg stress at any concentration inhibited the growth. The specific growth rates (*μ*) of *C. pyrenoidosa* cells under Hg^2+^ exposure were significantly lower than that of control (*p* < 0.05) after 24 h (Figure 1). The cells treated with 0.5 and 1 mg/L Hg^2+^ were not significantly different from each other, but significantly lower than the cells exposed to 0 and 0.05 mg/L Hg^2+^. 

### 3.2. Effects of Hg^2+^ on Pigments Content

**Figure 2:**
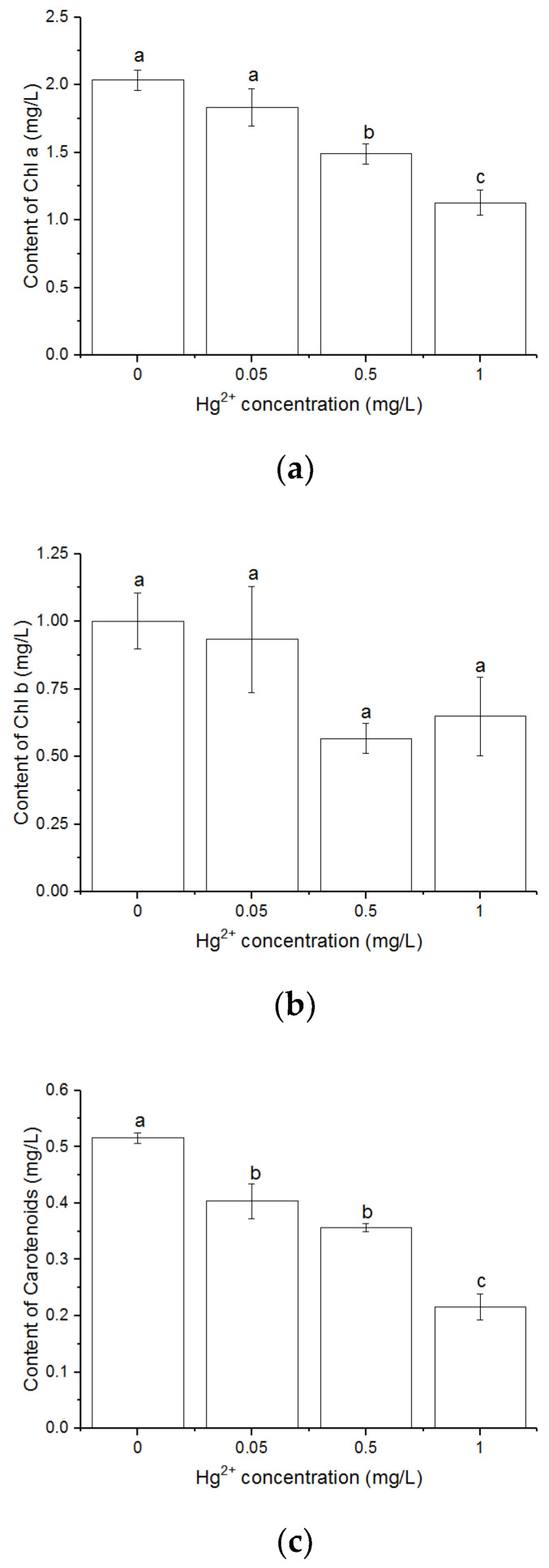
Contents of Chl *a* (**a**), Chl *b* (**b**) and carotenoids (**c**) after *C. pyrenoidosa* cells were exposed to various concentrations of Hg^2+^ for 24 h. Data are means ± S.E. (n = 3). Significant differences between different treatments were shown as different letters (*p* < 0.05, ANOVA, Duncan’s test).

The contents of Chl *a* (Figure 2a) and total carotenoids (Figure 2c) decreased with increasing Hg^2+^ concentration. The content of Chl *a* was significantly lower in the cultures with 0.5 and 1 mg/L Hg^2+^ than that of control (*p* < 0.05). The 1 mg/L Hg^2+^ showed serious effects on the content of Chl *a* and total carotenoids, which were significantly lower than other treatments. The Hg^2+^ treatments in the present experiment showed no significant decrease in the content of Chl *b* (Figure 2b).

### 3.3. Effects of Hg^2+^ on the Chlorophyll a Fluorescence Parameters

**Figure 3:**
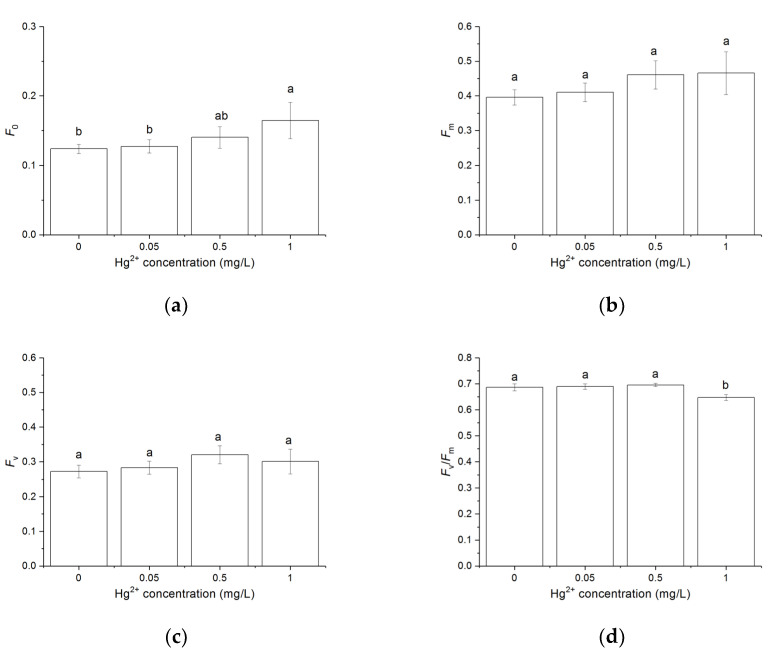
Effects of Hg^2+^ on the chlorophyll *a* fluorescence parameters. (**a**) The minimal fluorescence (*F*_0_). (**b**) The maximum fluorescence (*F*_m_). (**c**) The variable fluorescence *F*_v_. (**d**) The maximal photochemical efficiency of PSII (*F*_v_/*F*_m_). Measurements were carried out after *C. pyrenoidosa* cells were exposed to various concentrations of Hg^2+^ for 24 h. Data are means ± S.E. (n = 3). Significant differences between different treatments were shown as different letters (*p* < 0.05, ANOVA, Duncan’s test).

After the sample was dark-adapted for 5 min, *F*_0_ and *F*_m_ were determined through the application of saturation pulse. *F*_0_ increased with the increasing Hg^2+^ concentration and was significantly higher than control when the cells were treated with 1 mg/L Hg^2+^ (*p* < 0.05). *F*_m_ and *F*_v_ showed no significant difference between the different treatments. The treatment with 1 mg/L Hg^2+^ showed lower *F*_v_/*F*_m_ compared to other treatments (*p* < 0.05) (Figure 3).

### 3.4. Effects of Hg^2+^ on Quantum Yields of PSI and PSII

**Figure 4:**
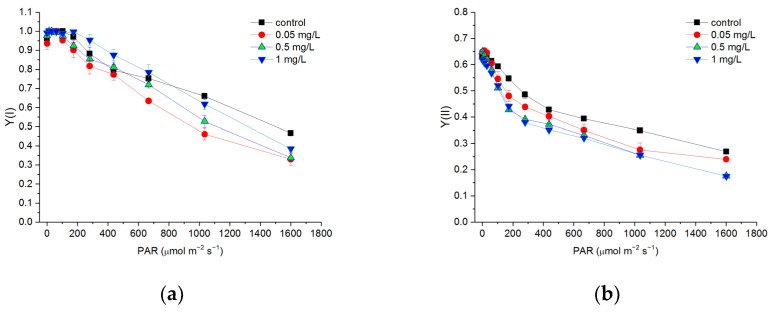
Rapid light curves (RLCs) of Y(I) (**a**) and Y(II) (**b**) after *C. pyrenoidosa* cells were exposed to various concentrations of Hg^2+^ for 24 h. Data are means ± S.E. (n = 3).

RLCs of Y(I) and Y(II) were measured in the light response reaction after the cells were exposed to various concentrations of Hg^2+^ for 24 h (Figure 4). Both RLCs of Y(I) and Y(II) decreased with increasing light intensity. Y(I) showed no significant difference between different treatments at a light intensity lower than 437 μmol m^−^^2^ s^−^^1^. With light intensity at 667 μmol m^−^^2^ s^−^^1^, the treatment with 0.05 mg/L Hg^2+^ showed lower Y(I) compared to other treatments (*p* < 0.05). With a light intensity at 1035 and 1601 μmol m^−^^2^ s^−^^1^, Y(I) of the cells treated with Hg^2+^ was significantly lower than that of the control (*p* < 0.05). Treatments with Hg^2+^ in the present experiment led to a significant decrease in Y(II) at a light intensity higher than 60 μmol m^−^^2^ s^−^^1^ (*p* < 0.05).

The complementary quantum yields of energy conversion in PSI and PSII of algal cells untreated and treated with Hg^2+^ for 24 h are shown in Table 1. In the cells treated with Hg^2+^, Y(I) and Y(NA) were significantly lower than that in the control (*p* < 0.05). In contrast, Y(ND) was significantly higher than that of the control (*p* < 0.05). Y(II) decreased with an increasing concentration of Hg^2+^, while Y(NPQ) increased with an increasing Hg^2+^ concentration and was significantly higher than the control when the cells were treated with 0.5 and 1 mg/L Hg^2+^ (*p* < 0.05). The Y(NO) of the cells treated with Hg^2+^ was significantly higher than that of the control (*p* < 0.05).

### 3.5. Effects of Hg^2+^ on Electron Transport Rates of PSI and PSII

**Figure 5:**
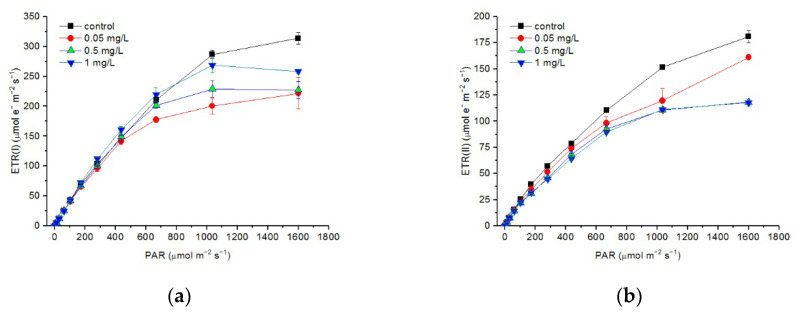
Rapid light curves (RLCs) of ETR(I) (**a**) and ETR(II) (**b**) after *C. pyrenoidosa* cells were exposed to various concentrations of Hg^2+^ for 24 h. Data are means ± S.E. (n = 3).

In general, the RLCs of ETR(I) and ETR(II) decreased when the cells were exposed to Hg^2+^ (Figure 5). The RLCs of ETR(I) and ETR(II) did not show a significant difference between different treatments at a light intensity lower than 667 μmol m^−^^2^ s^−^^1^. At a light intensity higher than 667 μmol m^−^^2^ s^−^^1^, the ETR(I) of cells treated with Hg^2+^ was significantly lower than that of the control (*p* < 0.05). ETR(II) significantly decreased with an increasing Hg^2+^ concentration at a light intensity higher than 437 μmol m^−^^2^ s^−^^1^, while there was no significant difference between the treatments with 0.5 and 1 mg/L Hg^2+^ (*p* < 0.05).

The descriptive parameters derived through the fitting of RLC showed more detailed information on responses of ETRs in PSI and PSII to Hg^2+^ exposure and increasing light intensity (Table 2). The *I*_k_ of the RLCs of both ETR(I) and ETR(II) and the ETR_max_ of the RLCs of ETR(I) were significantly lower in the cells exposed to Hg^2+^ (*p* < 0.05). The *α* of the RLCs of ETR(I) did not show a significant difference between the different treatments. In contrast, the *α* of the RLCs of ETR(II) showed no significant difference between the cells exposed to 0.05 mg/L Hg^2+^ and the control and significantly decreased with an increasing Hg^2+^ concentration thereafter (*p* < 0.05). The ETR_max_ of the RLCs of ETR(II) decreased with an increasing Hg^2+^ concentration and were significantly lower when the cells were treated with Hg^2+^ at all concentrations in the experiment (*p* < 0.05).

### 3.6. Response of Cyclic Electron Flow (CEF) to Hg^2+^ Treatments

**Figure 6:**
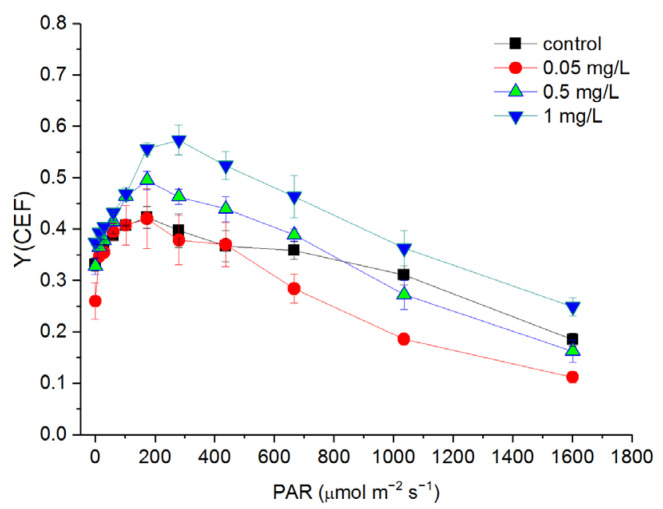
Rapid light curves (RLCs) of Y(CEF) after *C. pyrenoidosa* cells were exposed to various concentrations of Hg^2+^ for 24 h. Data are means ± S.E. (n = 3).

With increasing light intensity, Y(CEF) increased at a light intensity lower than 173 μmol m^−^^2^ s^−^^1^ and began to decrease at a light intensity higher than 280 μmol m^−^^2^ s^−^^1^ (Figure 6). The Y(CEF) of the cells treated with 0.05 mg/L Hg^2+^ showed no significant difference compared to the control at a light intensity lower than 667 μmol m^−^^2^ s^−^^1^ and decreased at a light intensity higher than 667 μmol m^−^^2^ s^−^^1^ (Figure 6). In general, the cells treated with 0.5 mg/L Hg^2+^ showed no significant difference in Y(CEF) compared to the control. The Y(CEF) of the cells treated with 1 mg/L Hg^2+^ was significantly higher than that of the control at a light intensity higher than 173 μmol m^−^^2^ s^−^^1^ (*p* < 0.05).

**Figure 7:**
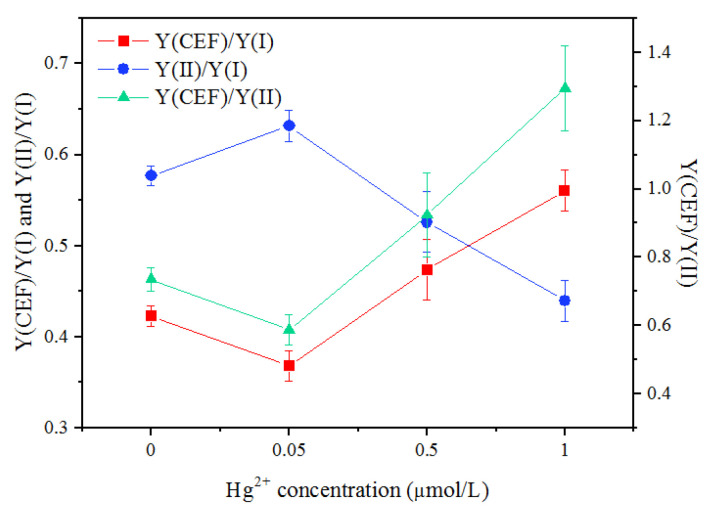
The ratios of Y(CEF)/Y(I), Y(CEF)/Y(II), and Y(II)/Y(I) after the cells were exposed to various concentrations of Hg^2+^ for 24 h. Data are means ± S.E. (n = 3). The quantum yields were detected after the last procedure of illumination at the highest intensity (1601 μmol m^−2^ s^−1^) during the light response reaction.

The change in the distribution of quantum yields between the two photosystems and the relationship between CEF and LEF under Hg exposure could be found in the change of Y(CEF)/Y(I), Y(II)/Y(I), and Y(CEF)/Y(II) (Figure 7). Y(CEF)/Y(I) and Y(CEF)/Y(II) slightly decreased after a 0.05 mg/L Hg^2+^ treatment, but largely increased with an increasing Hg^2+^ concentration (*p* < 0.05). The Y(II)/Y(I) generally decreased with an increasing Hg concentration, except for the treatment with 0.05 mg/L Hg^2+^. After the cells were exposed to 1 mg/L Hg^2+^ for 24 h, the Y(CEF) contributed larger to Y(I) than Y(II).

**Figure 8:**
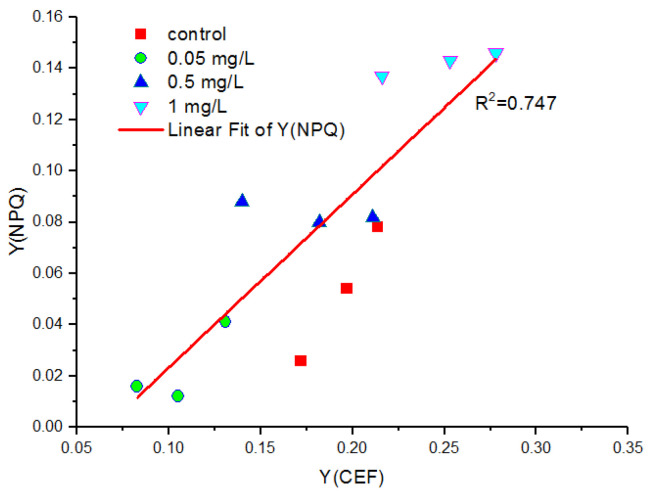
The correlations between Y(CEF) and Y(NPQ) after the cells were exposed to various concentrations of Hg^2+^ for 24 h. Data were detected after the last procedure of illumination at the highest intensity (1601 μmol m^−2^ s^−1^) during the light response reaction. All the data presented here were calculated from 3 replicated samples for each treatment.

The change of Y(CEF) and Y(NPQ), and their correlations after the cells were exposed to Hg^2+^ are shown in Figure 8. In general, Y(CEF) and Y(NPQ) increased with an increasing Hg concentration. Y(CEF) was positively correlated with Y(NPQ).

### 3.7. Binding Ability of Hg Ions with Photosystems

**Figure 9:**
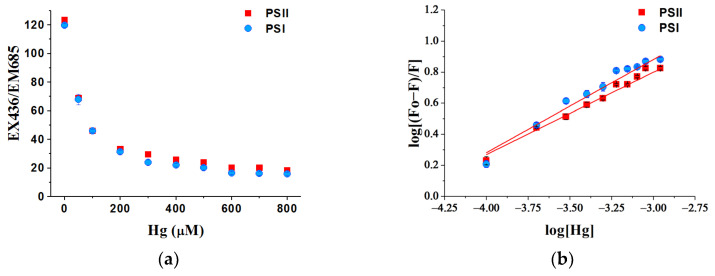
Quenching effects of Hg on fluorescence of photosystems particles isolated from *C. pyrenoidosa.* (**a**) The fluorescence intensity of photosystems at EX436/EM685 and the quenching of the fluorescence with Hg ions. (**b**) The equilibrium characteristics of binding process by fitting the fluorescence curves. Data are means ± S.E. (n = 3).

The fluorescence intensity of photosystems during the quenching with Hg ions is shown in Figure 9. Both the intensities of the fluorescence of the two photosystem particles posed a significant quenching process due to the addition of the Hg ions. 

More details of the quenching effects of Hg on the fluorescence are shown as fitting parameters in Table 3. The association constant (Ka) showed that Hg has a strong binding ability with the two photosystems. There were more binding sites (n) of Hg on PSII than on PSI.

## 4. Discussion

In the present study, the toxic effects of Hg^2+^ on the growth of *C. pyrenoidosa*, photosynthetic pigments content, and activities of PSI, PSII, and CEF were examined. Under Hg^2+^ exposure, the changes in quantum yields of PSI, PSII and CEF, the electron transport rates of PSI and PSII, and the relationship between Y(CEF) and Y(NPQ) were analyzed. The binding features of Hg ions and photosystem particles may explain the difference between the toxicity of Hg on PSII and PSI.

The main reason for the high toxicity of Hg^2+^ is its high affinity to thiol groups [46]. It was believed that the toxicity of heavy metals in photosynthesis is related to the binding abilities of metals to photosynthetic apparatus [47]. Therefore, the binding reaction between the photosystems and the heavy metal ions may explain their different toxicity. Through the quenching of fluorescence peaks, the migration characteristics of environmental pollutants and the complexation process of bioorganic substances were studied [48,49]. To understand the toxicity of Hg on photosystem apparatus and its characteristics different from other heavy metals, the binding abilities of heavy metals, e.g., Hg, Cd, and Ni, with photosystem particles were shown by the quenching effects of the fluorescence (Figure S3, Table S1). A strong binding ability between Hg and the two photosystems was found. As the association constant (Ka) showed that Hg has a strong binding ability with the two photosystems compared with Cd and Ni, this may explain the high toxicity of Hg to photosynthetic organisms. This result explained the high toxicity of Hg to the growth of *C. pyrenoidosa* and photosystems. The binding sites (n) of PSII and Hg were more than PSI. This was consistent with the results that Hg inhibited the quantum yield and electron transfer activity of PSII more seriously than those of PSI.

Algae and the chlorophyll *a* fluorescence data for trace element ecotoxicological trials were widely used as a global indicator [27,50]. Photosynthesis completes the conversion process of matter and energy, which is the basis of the growth of photosynthetic organisms and lives on earth. Therefore, it is necessary to study the physiology of photosynthesis and its regulation mechanism under stress [51]. Hg was reported to exert its toxic effect on growth [9,18], by inhibiting photosynthesis, nutrient uptake and metabolism [52]. 

The inhibition of Hg on the growth of green algal cells, which was due to the inhibition of Hg on photosynthesis, was confirmed in the present study. In some previous studies, the strong toxicity of Hg was demonstrated by the drastic inhibition of oxygen release and the inhibition of photochemical reactions observed in 30 μM Hg-treated *Chlamydomonas* [53]. Hg could damage the photosynthetic electron transport chain with multiple components [53,54]. It was observed that a decrease in the quantum yield of photosynthesis and the change in the photochemistry of PSII occurred in *Spirulina platensis* exposed to up to 20 µM Hg for 2 h [13]. Hg could bind to physiologically important organelles such as chloroplasts, affect photosynthesis, and cause the imbalance of reactive oxygen species concentrations [55]. An experiment with six algal species demonstrated that lower Hg concentrations (at the nanomolar level) were also toxic to the photosystem [56]. In contrast, the experiment with *Scenedesmus acutus* and *S. quadricauda* found that 0.15 mg/L Hg^2+^ inhibited the growth of the algae but no significant changes were observed in the contents of photosynthetic pigments and chlorophyll *a* fluorescence parameters [57]. The effects of Hg^2+^ on chlorophyll *a* fluorescence parameters were also found in the present study. *F*_m_ and *F*_v_ showed no significant difference between the different treatments. Hg^2+^ (1 mg/L) caused an increase in *F*_0_ and a decrease in *F*_v_/*F*_m_. The decrease in *F*_v_/*F*_m_ was due to the increase in *F*_0_. These results indicated that the openness of the PSII reaction centers decreased, and the functional state of PSII was affected by Hg [58]. The toxicity of Hg^2+^ might also be due to the inhibition of the active transport of nutrients, nitrogen starvation, and oxidative damage [59].

Similar to other heavy metals, Hg exposure could inhibit Chl *a* synthesis by substituting the central magnesium atom (Mg^2+^) [18,46], which consequently caused the inhibition of photosynthesis. The content of Chl *a* was also found to be seriously inhibited by a high concentration of Hg^2+^ (1 mg/L) in the present study, indicating the inhibition of Hg on Chl *a* synthesis. The function of chlorophylls can be impaired through the substitution of the Mg^2+^ ion in the chlorophyll molecule via toxic heavy metals, such as Cu, Cd, or Hg and the formation of heavy-metal-substituted chlorophylls eventually lead to serious damage to the whole photosynthetic process [60]. Accompanied by the substitution of metal ions by Hg in photosynthetic pigments, the loss of chlorophyll contents was also attributed to oxidative stress induced by Hg, and the decreased uptake of essential elements, such as Mn and K [61]. The inhibition of Hg on the activity of enzymes that catalyzed the chlorophyll biosynthesis was also confirmed [60]. Hg exposure did not show a significant effect on the content of Chl *b* in the experiment (Figure 2b). The content of total carotenoids was also inhibited by Hg (Figure 2c). As carotenoids aid in broadening the spectrum of PAR, and show a protective role as antioxidants [62,63], the accumulation of carotenoids may act as a protective mechanism for coping with chlorophyll deficit and a decrease in LEF [51,64]. The decrease in the content of total carotenoids under Hg exposure will lead to the inhibition of photosynthesis and damage to photosynthetic apparatus. 

These inhibitions and the damage of PSI and PSII were confirmed from the chlorophyll fluorescence and P700^+^ data in the cells treated with Hg^2+^. Photosynthetic electron transfer occurs in the thylakoid membrane of chloroplasts and is related to PSI, PSII and other electron carriers [51]. Many sites in the photosynthetic membrane: the donor side and acceptor side of PSI, the core of PSI [65], and especially PSII [12,54], are highly sensitive to Hg. PSII was shown to be the most sensitive target to Hg including its donor and acceptor sides [22,55]. However, the toxicity of Hg to PSI and PSII activities has rarely been analyzed simultaneously. The Dual-PAM-100 system shows its advantage in simultaneous measurements of chlorophyll fluorescence and P700^+^ absorbance changes, reflecting the physiological state of PSII and PSI at the same time [23,24]. It was introduced in the present study to detect the physiological response of photosynthetic apparatus to environmental stress as in some studies [33,40,41], which will be an accurate, rapid and efficient tool for toxicity bioassays. A few studies showed the data detected by the Dual-PAM-100 system for analysis of PSI and PSII activities under Hg treatment. With the increase in Hg concentrations, *F*_v_/*F*_m_, Y(II), Y(I), and Y(NPQ) showed a downward trend, while Y(ND) and Y(NO) displayed an upward trend in the leaves of *Brassica campestris* [66]. Hg^2+^ decreased the quantum yield and ETRs of PSI and PSII, whereas it increased the limitation of the donor sides in the aquatic plant *Microsorum pteropus* [12].

Similar to some previous studies [12,18], the toxic effects of Hg^2+^ on the activities of both PSII and PSI were shown in the present study. PSII was more sensitive to Hg^2+^ than PSI, indicated by less of a decrease in the RLCs of Y(I) (Figure 4) and the value of Y(I) at the highest intensity (1601 μmol m^−2^ s^−1^) during the light response reaction (Table 1). The lesser inhibition of Hg on PSI could also be derived from the RLCs and fitting curves of ETR(I) (Figure 5). In contrast to Y(I) and ETR(I), Y(II) and ETR(II) significantly decreased with an increasing Hg^2+^ concentration. This was in accordance with some studies showing that PSI is less affected than PSII under Hg stress [18,67]. Y(I) and ETR(I) showed no significant difference between the different treatments at a light intensity lower than 437 μmol m^−^^2^ s^−^^1^ ( Figure 4a and Figure 5a), mainly due to the enhancement of Y(CEF) under the Hg treatments (Figure 6). These results suggest less sensitivity of PSI to Hg and increasing irradiation as in some other heavy metal exposure [26]. The lesser sensitivity of PSI to increasing light intensity under Hg stress was also reflected from the descriptive parameters of the RLC of ETR: *a*, *I*_k_, ETR_max_. Where the *α* of the RLCs of ETR(I) did not show a significant difference between the different treatments but significantly decreased with an increasing Hg^2+^ concentration as for PSII. The ETR_max_ of PSI was higher than that of PSII and did not show a significant difference between the cells treated with Hg^2+^. This suggested that Hg^2+^ showed no more serious inhibition on ETR(I), and PSI showed tolerance to Hg stress. However, the descriptive parameters of the RLC of PSII kept decreasing with an increasing Hg^2+^ concentration, indicating serious inhibition of Hg^2+^ on PSII. The inhibition of Hg^2+^ on PSII could be more directly from the decrease in Y(II) and the increase in Y(NO). Y(NO) represents excess PSII energy that is dissipated via non-regulated processes and presented as a good indicator of PSII damage [25,40].

In general, heavy metals disturb the function of oxygen-evolving complexes and damage the proteins on the oxidation (donor) and PSII acceptor sides [68,69], and PSII was proposed to be more sensitive to heavy metal stress than PSI [60]. Moreover, it was reported that the most sensitive site of the metal inhibitor was located on the oxidation side of PSII, where a reversible inhibition of Tyrz (the redox-active tyrosine residue of the D1 protein) takes place [70]. As for the treatment with Cd, research had found that Cd inhibited PSII at the molecular level, while PSI activity was less affected [71]. Hg can strongly bind with thiol groups on the receptor side and donor side of PSII, interfering with the function of PSII [22]. Hg exerted its toxicity on the donor side of PSII by disturbing the chloride binding and/or function [72]. Hg also posed high toxicity to the reducing side of PSII, disturbing the electron transportation from PSII to PSI [73]. By using the Stern–Volmer method to analyze the mercury fluorescence quenching effect in the green alga, the previous study indicated the possibility of four Hg binding sites in the PSII complex, suggesting the relationship between Hg inhibition of PSII and these Mn active sites in the oxygen-evolving complex [74]. The binding reaction between photosystems and Hg in the present study found that the binding sites (n) of PSII and Hg were more than PSI, which may explain the reason that Hg inhibited the quantum yield and electron transfer activity of PSII more seriously than those of PSI.

The significant increase in Y(ND) under Hg^2+^ stress suggested that PSI was still well-regulated under Hg^2+^ treatments in the experiment, and the decrease in the photochemistry of PSI is owing to the inefficient electron donor and inefficient light absorption of PSII [12]. Y(NA) decreased after exposure to Hg^2+^, mainly due to the activation of CEF under the treatment of Hg^2+^. The decrease in Y(NA) indicated the lack of acceptor side limitation, which will prevent PSI from over-reduction of the acceptor side and will not lead to restriction of PSI activity [75]. These results were in agreement with the suggestion that CEF was stimulated as an important mechanism for preventing acceptor side limitation of PSI [76,77]. These results indicated the important role of CEF in the protection of PSI under Hg stress. 

The significant contribution of CEF to the quantum yield of PSI could also be found in the change in the distribution of quantum yields between the two photosystems, indicated by the ratios of Y(CEF)/Y(I), Y(II)/Y(I), and Y(CEF)/Y(II). The increase in Y(CEF)/Y(II) increased the enhancement of the quantum yield of CEF and the inhibition of LEF under Hg^2+^ stress. Y(CEF) contributed larger to Y(I) than Y(II) after the cells were exposed to 1 mg/L Hg^2+^ for 24 h, indicated by the increase in Y(CEF)/Y(I) and the decrease in Y(II)/Y(I) (Figure 7).

The activation of CEF under Hg^2+^ stress found in the present study confirmed the role of CEF in its contribution to the formation of the trans-thylakoid membrane proton gradient (ΔpH) [75,78], which was helpful for NPQ [77]. This was confirmed by the increase in Y(CEF) and Y(NPQ) due to Hg^2+^ treatment and the positive correlation between them (Figure 8). The increase in Y(NPQ) led to a dissipation of excessive excitation energy into harmless heat [33], showing the protective role of the photosynthetic apparatus. The activation of CEF and NPQ was the important mechanism to protect photosynthetic apparatus under Hg^2+^ stress.

## 5. Conclusions

In summary, inhibition of Hg^2+^ on the cell’s growth and pigment synthesis was found. Chl *a* was sensitive to Hg^2+^ exposure. The activities of PSII and PSI of *C. pyrenoidosa* to Hg^2+^ stress were measured simultaneously. PSI was more resistant to Hg^2+^ than PSII, indicated by a smaller decrease in the RLCs of Y(I) and ETR(I). The serious inhibition of Hg^2+^ on PSII could be derived from the significant decrease in Y(II) and increase in Y(NO). Y(NO) presented as a good indicator of PSII damage under Hg^2+^ stress. Hg^2+^ had a more inhibitory effect on the efficiency of PSII to use light energy and the maximum electron transport rate than that of PSI. The stimulation of CEF under Hg^2+^ stress was essential for the stability and protection of PSI and helpful for the conduction of non-photochemical quenching to protect the photosynthetic apparatus. The strong binding ability of Hg^2+^ and the two photosystems may explain the high toxicity of Hg and the different toxicity of Hg on PSII and PSI.

## Figures and Tables

**Table 1:** The complementary quantum yields of energy conversion in PSI and PSII after exposure to various concentrations of Hg_2+_ for 24 h. Data were detected after the last procedure of illumination at the highest intensity (1601 μmol m^−2^ s^−1^) during the light response reaction. Data are means ± S.E. (n= 3), and data followed by different letters in the same column are significantly different (*p* < 0.05, ANOVA, Duncan’s test).

Hg^2+^ Concentration (mg/L)	Quantum Yields in PSI			Quantum Yields in PSII		
	Y(I)	Y(ND)	Y(NA)	Y(II)	Y(NO)	Y(NPQ)
0	0.467 ± 0.014 ^a^	0.342 ± 0.035 ^b^	0.178 ± 0.037 ^a^	0.270 ± 0.008 ^a^	0.653 ± 0.001 ^c^	0.040 ± 0.014 ^c^
0.05	0.330 ± 0.040 ^b^	0.670 ± 0.050 ^a^	0.040 ± 0.024 ^b^	0.240 ± 0.001 ^b^	0.790 ± 0.029 ^a^	0.023 ± 0.009 ^c^
0.5	0.338 ± 0.021 ^b^	0.660 ± 0.039 ^a^	0.027 ± 0.019 ^b^	0.176 ± 0.002 ^c^	0.740 ± 0.004 ^ab^	0.086 ± 0.003 ^b^
1	0.384 ± 0.009 ^b^	0.603 ± 0.037 ^a^	0.035 ± 0.022 ^b^	0.175 ± 0.005 ^c^	0.693 ± 0.005 ^b^	0.137 ± 0.005 ^a^

**Table 2:** Descriptive parameters of the rapid light curves (RLCs) of ETR(I) and ETR(II). *A*, the initial slope of the RLC; ETR_max_, maximal electron transport rate; *I*_k_, the sub-saturation irradiance. The measurements were carried out after exposure to various concentrations of Hg^2+^ for 24 h. Data are means ± S.E. (n = 3), and data followed by different letters in the same column are significantly different (*p* < 0.05, ANOVA, Duncan’s test).

Hg^2+^ Concentration (mg L^−1^)	Parameters of RLCs of ETR(I)			Parameters of RLCs of ETR(II)		
	*I*_k_ (μmol Photon m^−2^ s^−1^)	*α* (e^−^ Photon^−1^)	ETR_max_ (μmol e^−^ m^−2^ s^−1^)	*I*_k_ (μmol Photon m^−2^ s^−1^)	*α* (e^−^ Photon^−1^)	ETR_max_ (μmol e^−^ m^−2^ s^−1^)
0	765.90 ± 64.41 ^a^	0.440 ± 0.025 ^a^	333.52 ± 12.28 ^a^	901.65 ± 74.58 ^a^	0.230 ± 0.003 ^a^	207.42 ± 17.00 ^a^
0.05	549.97 ± 89.22 ^b^	0.430 ± 0.028 ^a^	231.13 ± 24.95 ^b^	715.70 ± 8.50 ^b^	0.232 ± 0.001 ^a^	165.85 ± 1.55 ^b^
0.5	493.92 ± 22.89 ^b^	0.477 ± 0.008 ^a^	235.62 ± 12.30 ^b^	564.00 ± 10.78 ^b^	0.211 ± 0.002 ^b^	118.98 ± 1.36 ^c^
1	552.00 ± 14.93 ^b^	0.489 ± 0.024 ^a^	277.20 ±15.40 ^b^	588.58 ± 24.41 ^b^	0.202 ± 0.003 ^c^	118.75 ± 3.60 ^c^

**Table 3:** Fitting parameters of quenching curves of the fluorescence of photosystems particles by titration of Hg. Data are means ± S.E. (n = 3).

Heavy Metal	PSI Particles		PSII Particles	
	Ka (×10^4^ M^−1^)	n	Ka (×10^4^ M^−1^)	n
Hg	2.68 ± 0.19	0.69 ± 0.09	2.72 ± 0.12	0.79 ± 0.07

## Data Availability

The datasets used or analyzed during the current study are available from the corresponding author on reasonable request.

## Supplementary Materials

The following supporting information can be downloaded at: https://www.mdpi.com/article/10.3390/toxics10080455/s1, Figure S1: Cell growth of *Chlorella pyrenoidosa* at various Hg concentrations expressed as the optical density at 680 nm (OD_680_), Figure S2: A typical experimental result obtained with Dual-PAM-100. The sample was dark-adapted for 5 min before measurement. P700^+^ absorbance changes and chlorophyll *a* fluorescence were detected through the application of saturation pulse. Figure S3: Quenching effects of Cd and Ni on fluorescence of photosystems particles isolated from *C*. *pyrenoidosa*, Table S1: Fitting parameters of quenching curves of the fluorescence of photosystems particles by titration of heavy metals.
