# Supplementary material for: The Binding Ability of Mercury (Hg) to Photosystem I and II Explained the Difference in Its Toxicity on the Two Photosystems of Chlorella pyrenoidosa
Source: Toxics. 2022 Aug 6;10(8):455. doi: 10.3390/toxics10080455 (PMC9416214; doi:10.3390/toxics10080455)
Supplement: Supplementary file 1 [file toxics-10-00455-s001.zip › toxics-1845707-proof resubmitted supplementary.pdf]

toxics

MDPI

Supplementary Materials: The Binding Ability of Mercury (Hg) to Photosystem I and II Explained the Difference in Its Toxicity on the Two Photosystems of *Chlorella pyrenoidosa*

Shuzhi Wang, Jia Duo, Rehemanjiang Wufuer, Wenfeng Li and Xiangliang Pan

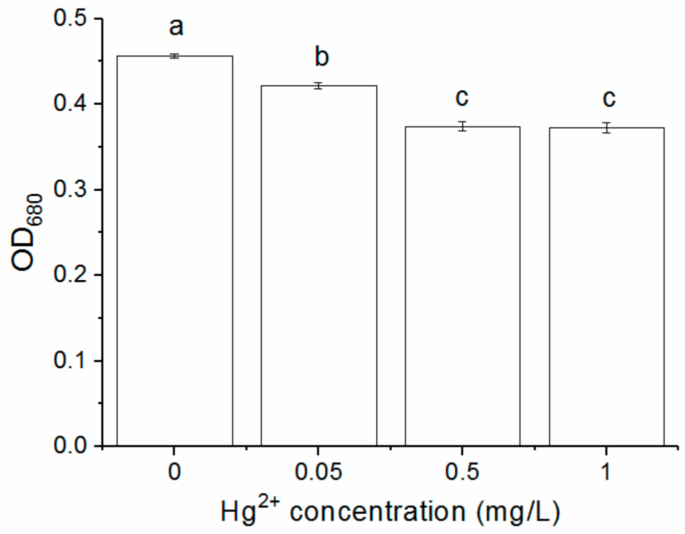

Figure S1. Cell growth of *Chlorella pyrenoidosa* at various Hg concentrations expressed as the optical density at 680 nm (OD_{680}). Cells were cultured in BG-11 medium with various concentrations of Hg^{2+} for 24 h. Data are means ± S.E. (n = 3). Significant differences between different treatments were shown as different letters ($p < 0.05$, ANOVA, Duncan's test).

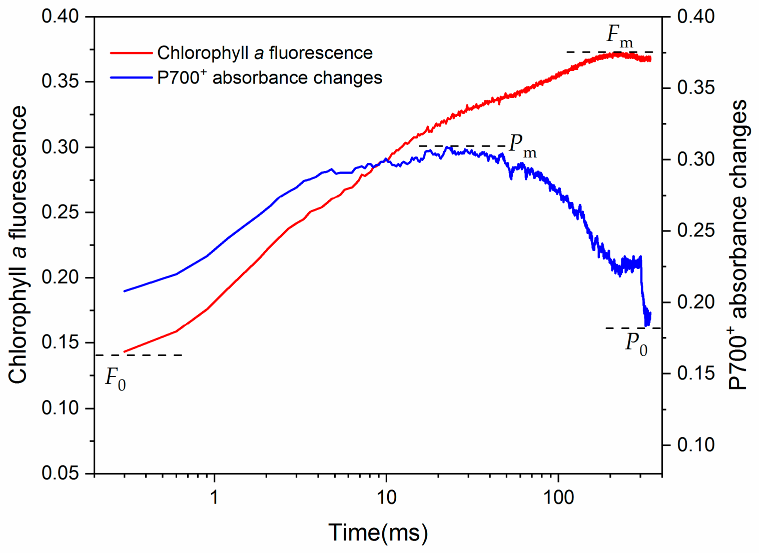

Figure S2. A typical experimental result obtained with Dual-PAM-100. The sample was dark-adapted for 5 min before measurement. $P700^+$ absorbance changes and chlorophyll *a* fluorescence were detected through the application of saturation pulse. The minimal fluorescence after dark-adaptation (F_0), the maximum fluorescence (F_m), and the maximal change in $P700^+$ signal (P_m) were determined through application of saturation pulse. The $P700^+$ absorbance changes were detected by the application saturation pulse after far-red pre-illumination to determine P_m. After the saturation pulse the minimal P700+ signal (P_0) was measured.

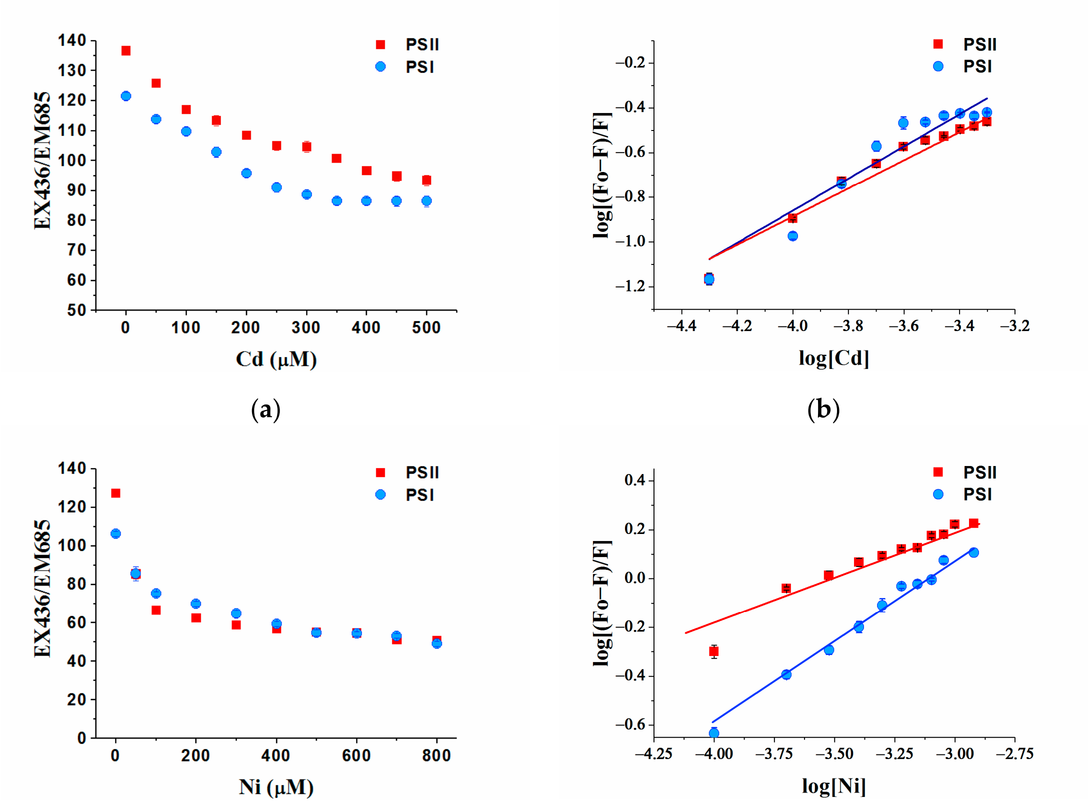

(**a**) (**b**)

(c) (d)

Figure S3. Quenching effects of Cd and Ni on fluorescence of photosystems particles isolated from *C. pyrenoidosa*. (**a** and **c**) The fluorescence intensity of photosystems at EX436/EM685 and the quenching of the fluorescence with Cd or Ni ions. (**b** and **d**) The equilibrium characteristics of binding process by fitting of the fluorescence curves. Data are means ± S.E. (n = 3).

Table S1. Fitting parameters of quenching curves of the fluorescence of photosystems particles by titration of heavy metals.

Heavy metal	PSI particles		PSII particles	
	Ka ($\times 10^4$ M^{-1})	n	Ka ($\times 10^4$ M^{-1})	n
Hg	2.68 ± 0.19	0.69 ± 0.09	2.72 ± 0.12	0.79 ± 0.07
Cd	2.12 ± 0.16	0.57 ± 0.08	1.97 ± 0.07	0.78 ± 0.05
Ni	2.09 ± 0.13	0.46 ± 0.06	1.26 ± 0.09	0.37 ± 0.04

Note: Data are means ± S.E. (n = 3).
